# Reasons for gender inequities in invasive electrophysiology: a survey on family issues and career paths of female and male electrophysiology fellows in Germany

**DOI:** 10.1093/ehjopen/oeae070

**Published:** 2024-08-20

**Authors:** Johanna Mueller-Leisse, Henrike Aenne Katrin Hillmann, Joerg Eiringhaus, Eleonora Angelini, Nizar Karfoul, Stephan Hohmann, David Duncker

**Affiliations:** Hannover Heart Rhythm Center, Department of Cardiology and Angiology, Hannover Medical School, Carl-Neuberg-Street 1, 30625 Hannover, Germany; Hannover Heart Rhythm Center, Department of Cardiology and Angiology, Hannover Medical School, Carl-Neuberg-Street 1, 30625 Hannover, Germany; Hannover Heart Rhythm Center, Department of Cardiology and Angiology, Hannover Medical School, Carl-Neuberg-Street 1, 30625 Hannover, Germany; Hannover Heart Rhythm Center, Department of Cardiology and Angiology, Hannover Medical School, Carl-Neuberg-Street 1, 30625 Hannover, Germany; Hannover Heart Rhythm Center, Department of Cardiology and Angiology, Hannover Medical School, Carl-Neuberg-Street 1, 30625 Hannover, Germany; Hannover Heart Rhythm Center, Department of Cardiology and Angiology, Hannover Medical School, Carl-Neuberg-Street 1, 30625 Hannover, Germany; Hannover Heart Rhythm Center, Department of Cardiology and Angiology, Hannover Medical School, Carl-Neuberg-Street 1, 30625 Hannover, Germany

**Keywords:** Gender disparities, Gender equity, Women in EP, Radiation exposure during pregnancy, Career paths, Invasive EP

## Abstract

**Aims:**

Female physicians are underrepresented in invasive electrophysiology (EP) for multiple reasons. Despite an increasing focus on the topic, it is unclear what aspects are predominant.

**Methods and results:**

We conducted a survey on career paths of current or former EP fellows in Germany to elucidate how gender and family affected their careers. 231 fellows (24.2% female) were invited. 110 participants completed the survey (30.9% female, mean age 41.0 ± 5.0 years, and 79.1% with children). Female and male participants with children reported similar career goals and achievements before parenthood, but afterwards women changed their career paths more often. Major reasons were personal priorities followed by lack of flexibility at work and at home. Women covered the majority of childcare. At the time of the survey, 80.0% of women and 96.4% of men with a former career goal of invasive EP were active in invasive EP. Independent of age, women were in lower-level positions, had accomplished fewer professional achievements, were less satisfied with their work and had fewer children. 56.5% of women did not feel supported by their employers regarding family issues. 82.6% reported there was no satisfactory day care. 69.6% were unable to continue to follow their career during pregnancy, mostly due to restrictions by employers (75.0%). Dedicated policies for pregnant workers or support programmes were scarce.

**Conclusion:**

Beside the distribution of childcare at home, lack of flexibility and support by employers as well as working and fluoroscopy restrictions during pregnancy hamper women in EP and should be addressed.

## Graphical Abstract

**Graphical abstract:**
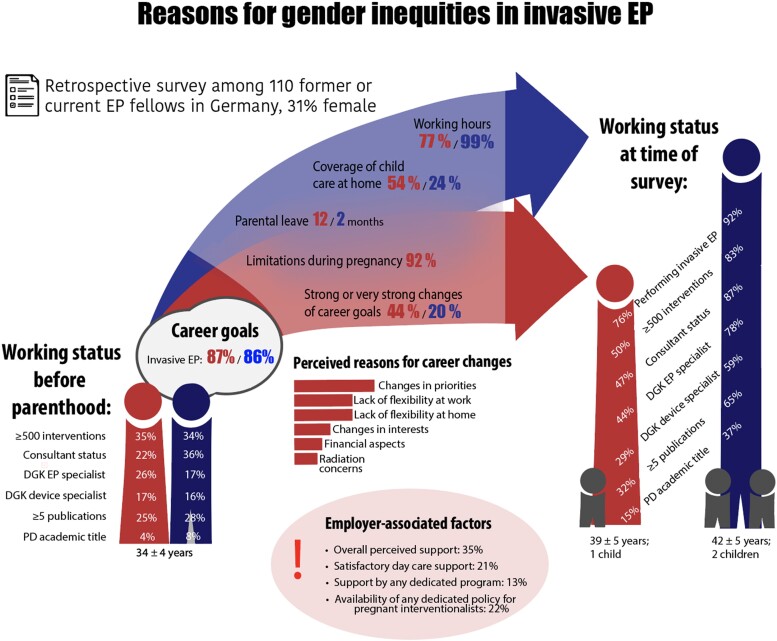
DGK, German Cardiac Society; EP, electrophysiology; DGK EP specialist, Part A of the DGK certification ‘Special Rhythmology’; DGK device specialist, Part B of the DGK certification ‘Special Rhythmology’; PD, ‘Privatdozent’ (associate professor).

## Introduction

Although the number of female physicians is growing, female physicians are still underrepresented in interventional cardiology including cardiac electrophysiology (EP), and especially in leading positions and research workforces worldwide.^1–6^ In Germany, for example, only ∼21% of consultant physicians and 2% of leaders in EP are female.^3^ Within the German Cardiac Society, 21% of working group nuclei members and none of the executive board members in EP are female.^2^ Cardiology societies are increasingly aware of this inequity, and several national and international surveys aimed to evaluate the career choices of female cardiologists to approach the issue.^7–12^

The underlying reasons are multifactorial and show similarities among different countries. They were shown to include divergent interests and ambitions to begin with, but also gender roles, discrimination, sexism, concerns about radiational exposure, and family concerns.^7–19^

The present study explores the causes for gender inequities in invasive EP more precisely. We conducted a survey targeting female and male study participants with a former or current career goal of invasive EP and similar starting points in terms of professional interests and ambitions, including research, at an earlier point in their career. The aim was to elaborate how gender, family, and working conditions affected their career paths.

## Methods

### Study population

The study population consisted of female and male physicians who were currently participating in dedicated educational programmes for invasive EP in Germany, or who had participated in such programmes in the past. The programmes provide exclusive training in the field of EP and are meant to support aspiring young doctors at the beginning of their EP careers. Participants are carefully selected. 231 current and former fellows (24.2% female) were invited via email or postal letter. The survey was conducted between September and October 2023.

### Questionnaire

An anonymous online questionnaire was designed using the SurveyMonkey platform (SurveyMonkey, San Mateo, CA, USA). It consisted of 37 questions: 10 questions on current work and family status, 20 questions on working status, and career goals before parenthood and on the impact of children on working conditions, ambitions and career, as well as 7 questions on the impact of pregnancy itself, using conditional branching depending on the respondent’s answers. Question format was multiple choice, Lickert scale or check boxes. Leading position was defined as consultant status, head of department or subdivision, or head of research group. The detailed questionnaire is displayed in the Supplementary material.

### Statistical analysis

Statistical analysis was performed using SPSS version 29 (IBM Corporation, Armonk, NY). Only fully completed answers with available participants’ consent were included in the analysis. Categorical variables are presented as numbers and percentages, continuous variables as means with standard deviations or median with range, as appropriate.

For comparisons, *χ*^2^ test or binary/multinomial logistic regression analysis was used for categorical variables, as appropriate. Linear or logistic regression analysis or Mann–Whitney *U* test was used for continuous variables. Bonferroni correction was applied as appropriate. A *P*-value <0.05 was considered statistically significant.

## Results

### General characteristics

One hundred and ten participants completed the survey, 30.9% female, mean age 41.0 ± 5.0 years, 79.1% had children. Women were younger than men (39.2 ± 5.0 vs. 41.8 ± 5.4 years, *P* = 0.012), were less likely to have children (67.6% vs. 84.2%, *P* = 0.045) and had fewer children (1 vs. 2 in median, *P* = 0.027 after adjustment for age).

### Current work status and professional achievements

Work and family status at the time of the survey are shown in *Table 1*. 42.7% of participants were working in a university hospital, 46.4% in a non-university hospital, and 10.9% in a private practice, with no significant gender differences. 88.2% of women and 100% of men were doing interventions at the time of the survey (*P* = 0.002): 76.5% vs. 92.1% were active in invasive EP (*P* = 0.043), 44.1% vs. 67.1% in device surgery (*P* = 0.040), and 5.9% vs. 38.2% in interventional cardiology (*P* = 0.007; each after adjustment for age). Considering only participants with children who had stated a career goal of invasive EP before parenthood, 80.0% of women and 96.4% of men were active in invasive EP (*P* = 0.04). Women more often worked reduced hours (55.6% of women vs. 6.6% of men; *P* < 0.001). Among women with reduced working hours, the majority (63.2%) still worked at least 75% of full-time equivalents.

**Table 1:** Current work and family status

	*All*	*Women*	*Men*	*P-value*
*n (%)*	110	34 (30.9)	76 (69.1)	
*Age* (*years*)	41.0 ± 5.0	39.2 ± 5.0	41.8 ± 5.4	0.012
*Children, n (%)*	87 (79.1)	23 (67.6)	64 (84.2)	0.045
*Number of children, median (range)*	2 (0–4)	1 (0–3)	2 (0–4)	0.027*
*Current work place*				0.765
*University hospital*	47 (42.7)	16 (47.1)	31 (40.8)	
*Non-*u*niversity hospital*	51 (46,4)	14 (41.2)	37 (48.7)	
*Private practice*	12 (10.9)	4 (11.8)	8 (10.5)	
*Working reduced hours, n (%)*	24 (21.8)	19 (55.9)	5 (6.6)	<0.001
*75–99% of full-time work*	15 (13.6)	12 (35.3)	3 (3.9)	
*50–74% of full-time work*	7 (6.4)	5 (14.7)	2 (2.6)	
*< 50% of full-time work*	2 (0.9)	2 (5.8)	0 (0)	
*Interventional work*				
*Any interventional activity, n (%)*	106 (96.4)	30 (88.2)	76 (100)	0.002
*Invasive electrophysiology, n (%)*	96 (87.3)	26 (76.5)	70 (92.1)	0.043*
*Device surgery, n (%)*	66 (60.0)	15 (44.1)	51 (67.1)	0.040*
*Interventional cardiology, n (%)*	31 (28.2)	2 (5.9)	29 (38.2)	0.007*

DGK: German Cardiac Society.

^*^Significant after adjustment for age.

In terms of clinical experience and professional achievements, independent of age, women had done fewer overall invasive procedures compared to men (*n* ≥ 500 interventions in 50.0% vs. 82.9%, *P* = 0.007), and had less often accomplished parts A (invasive EP) or B (devices) of the ‘Special Rhythmology’ certification of the German Cardiac Society (DGK): 44.1% vs. 77.6% (*P* = 0.005) and 29.4% vs. 59.2% (*P* = 0.043), respectively. Women had published fewer articles as junior or senior author (*n* ≥ 15 publications in 8.8% vs. 32.9%, *P* = 0.045) and had less often achieved habilitation, referring to the German academic degree ‘Privatdozent’ meaning associate professor, or professor (14.7% vs. 36.8%; *P* = 0.032), independent of age. Women and men were in leading positions in 47.1% and 86.8%, respectively (*P* < 0.001 after adjustment for age).

**Figure 1:**
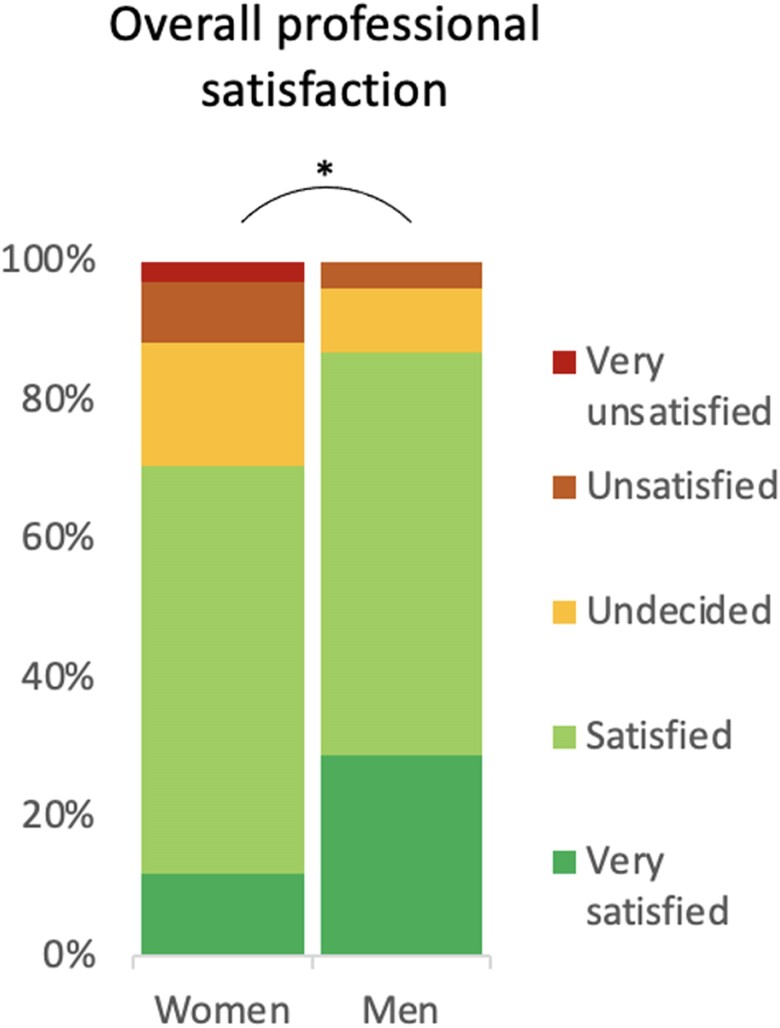
Satisfaction at work.

While most women and men were overall satisfied with their work (70.6% and 86.8%, respectively), there was still a significant difference between genders, as women were less often ‘very satisfied’ (11.8% vs. 28.9%) and more often ‘unsatisfied’ (8.8% vs. 3.9%) or ‘very unsatisfied’ (2.9% vs. 0%); *P* = 0.011 (*Figure 1*).

### Status before parenthood

Comparing the starting points of both genders before parenthood (at mean age 33.5 ± 3.6 years), there were no significant differences in career goals, which included invasive EP in 87.0% of women and 85.9% of men. All women and men had been working full-time. More participants were working at a university hospital before compared to after parenthood (65.5% vs. 42.7%; *P* = 0.002; with no difference between genders).

Women and men had achieved qualifications to a similar extent, including completion of cardiology training in 43.5% vs. 37.5%, parts A (invasive EP) and B (devices) of the DGK ‘Special Rhythmology’ certification in 26.1% vs. 17.2% and in 17.4% vs. 15.6%, respectively. There was no significant difference in the numbers of invasive procedures that had been performed by women and men before parenthood, 34.8% and 34.4% having performed ≥500 procedures and 30.4% and 25.0% 100–499 procedures. There was also no difference between genders in terms of academic titles or research activity. The majority of women and men had published 1–4 articles (47.8% and 48.4%, respectively) or 5–9 articles (17.4% and 14.1%), in peer-reviewed journals as a first or last author. 21.7% of women and 35.9% of men were already in leading positions before parenthood (*P* = 0.211).

**Figure 2:**
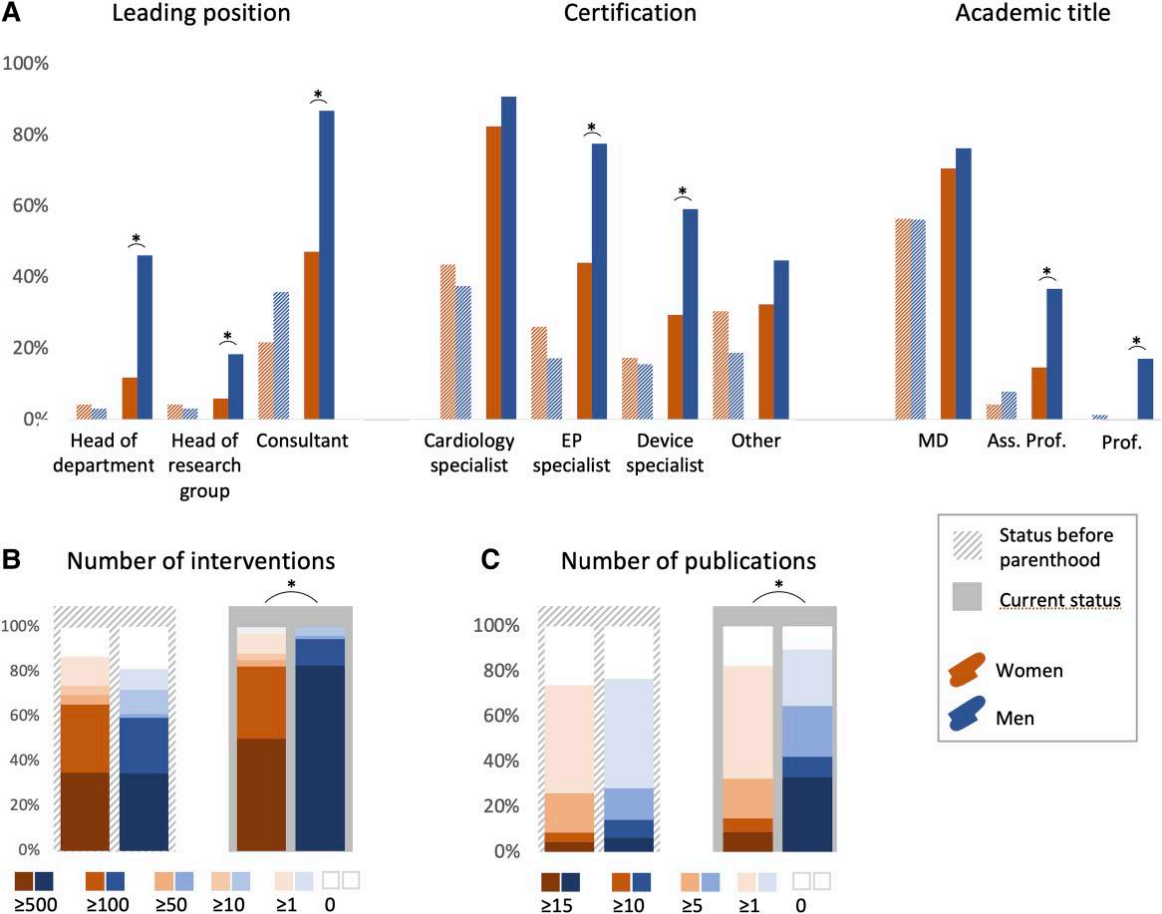
Comparison of professional achievements between genders before parenthood and at time of survey (working position, certifications and academic titles, (*A*); number of interventions performed, (*B*); nunber of pubmed-listed publications, (*C*). EP, electrophysiology; EP specialist refers to Part A of the certification ‘Special Rhythmology’ by the German Cardiac Society (DGK); Device specialist refers to Part B of the certification ‘Special Rhythmology’ by DGK; MD, Medical doctor, referring to the German academic degree Dr. med.; Ass. prof.: Associate professor, referring to the German academic degree PD, ‘Privatdozent’; asterisks indicate statistically significant differences (*P* < 0.05). After adjustment for age, the following differences remained statistically significant: Head of department, consultant, EP specialist, device specialist, ass. prof., number of interventions, number of publications.

*Table 2* shows the status before parenthood, *Figure 2* shows professional achievements, both before parenthood and at the time of the survey.

**Table 2:** Status before parenthood

	*All*	*Women*	*Men*	*P-value*
*n* (%)	87	23 (26.4)	64 (73.5)	
Age at first parenthood (years)	33.5 ± 3.6	33.6 ± 3.5	33.5 ± 3.7	0.545
Career goals				
*Invasive electrophysiology*	75 (86.2)	20 (87.0)	55 (85.9)	0.903
*Interventional cardiology*	22 (25.3)	3 (13.0)	19 (29.7)	0.115
*Research career*	37 (42.5)	7 (30.4)	30 (46.9)	0.171
Work place				0.308
*University hospital*	57 (65.5)	15 (65.2)	42 (65.6)	
*Non-*u*niversity hospital*	27 (31.0)	6 (26.1)	21 (32.8)	
*Private practice*	2 (2.3)	1 (4.3)	1 (1.6)	
Working reduced hours, *n* (%)	0	0	0	

DGK: German Cardiac Society.

### Changes in career paths after parenthood

**Figure 3:**
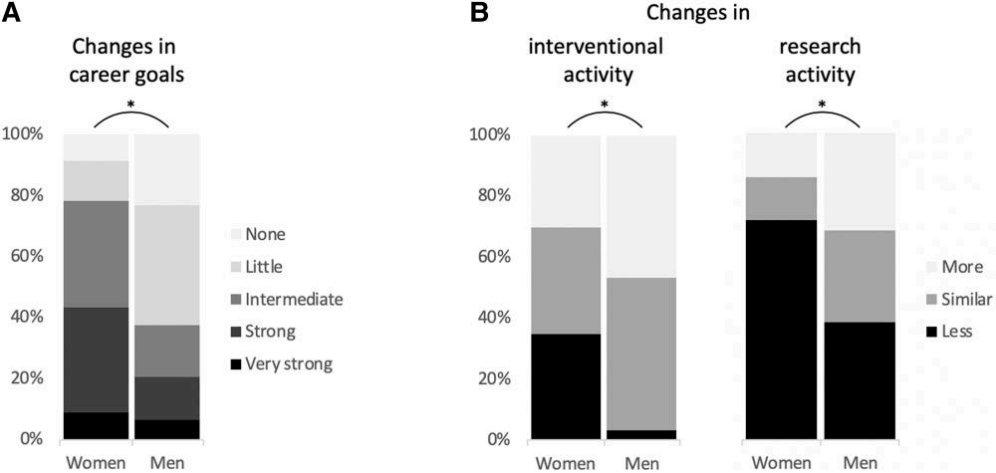
Comparison of career changes after parenthood between genders (changes in career goals, (*A*); changes in interventional and research activity, (*B*). Asterisks indicate statistically significant differences (*P* < 0.05).

**Figure 4:**
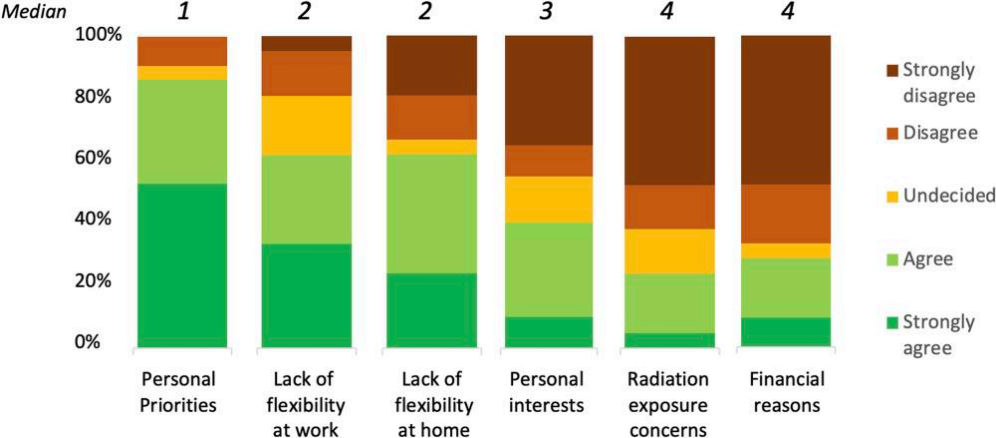
Reasons for career changes after parenthood reported by women.

Women reported to have changed their career goals after parenthood to a higher degree than men (*Figure 3*). Most women reported intermediate (34.8%) or strong (34.8%) changes, while most men reported no (23.4%) or little (39.1%) changes (*P* = 0.004). 36.1% of women and 3.4% of men reduced their interventional activity after parenthood (*P* = 0.034). Overall, 80.0% of women and 96.4% of men with a prior career goal of invasive EP before parenthood were active in invasive EP at the time of the survey (*P* = 0.021). 71.4% of women and 38.3% of men reduced their research activity after parenthood (*P* = 0.042). Reasons for career changes included changes in personal priorities reported by most women (52.4% ‘strongly agreed’ and 33.3% ‘agreed’) followed by a lack of flexibility at work (33.3% ‘strongly agreed’ and 28.6% ‘agreed’) and at home (23.8% ‘strongly agreed’ and 38.1% ‘agreed’). Changes in personal interests, radiation exposure or financial reasons were less important for most women (*Figure 4*). Reasons for career changes for both genders are shown in the Supplementary material.

### Factors at home and at work

73.9% of women and 85.7% of men felt supported by their families in terms of career goals and working hours (*P* = 0.052). Women reported to cover the majority of childcare at home (on average, they reported to cover 53.9 ± 21.1% of total childcare), while men did not (they reported to cover 24.2 ± 12.5% of total childcare) (*P* < 0.001). After child birth, women and men took off 11.0 ± 4.5 vs. 1.5 ± 1.2 months for parental leave (*P* < 0.001). After parental leave, women reduced their working hours to a higher extent than men (mean working hours 79.6 ± 16.2% vs. 98.5 ± 5.3% of full-time work, respectively; *P* < 0.001). Additional absences from work for childcare, such as child sick leave, were reported to comprise 6.0 ± 5.7 vs. 2.8 ± 2.8 days per year on average by women and men, respectively (*P* < 0.001).

Most women (56.5%) did not feel supported by their employers in terms of work-family conflicts, while most men felt supported (39.1%) or were undecided (25.0%; *P* = 0.188). 81.6% of employers offered some sort of day care for children, but only 21.1% of women and 52.0% of men stated these were satisfactory (*P* = 0.045). 13.0% of women and 3.1% of men received support by dedicated grants or programmes (*P* = 0.121). Employer and family factors are summarized in *Table 3*.

**Table 3:** Employer and family factors

	*All*	*Women*	*Men*	*P-value*
Parental leave (months)	3.9 ± 4.9	11.0 ± 4.5	1.5 ± 1.2	<0.001
Average working hours since parenthood (%)	94.5 ± 12.6	79.6 ± 16.2	98.5 ± 5.3	<0.001
Distribution of childcare at home (%)				
*Self*	32.1 ± 20.0	53.9 ± 21.1	24.2 ± 12.5	<0.001
*Partner*	59.4 ± 21.7	40.0 ± 21.4	66.3 ± 17.3	<0.001
*Other*	12.0 ± 16.1	13.0 ± 14	11.6 ± 17	0.310
Additional days absent for childcare (average/year)	3.7 ± 4.0	6.0 ± 5.7	2.8 ± 2.8	<0.001
Perceived support by family, Likert scale median (range)	2 (1–5)	2 (1–4)	1 (1–5)	0.052
*1: Strongly agree*	38 (44.2)	6 (26.1)	32 (50.8)	
*2: Agree*	33 (38.4)	11 (47.8)	22 (34.9)	
*3: Undecided*	9 (10.5)	5 (21.7)	4 (6.3)	
*4: Disagree*	4 (4.7)	1 (4.3)	3 (4.8)	
*5: Strongly disagree*	2 (2.3)	0	2 (3.2)	
Perceived support by employer/supervisors, Likert scale median (range)	3 (1–5)	4 (1–5)	3 (1–5)	0.188
*1: Strongly agree*	7 (8.0)	1 (4.3)	6 (9.4)	
*2: Agree*	26 (29.9)	7 (30.4)	19 (29.7)	
*3: Undecided*	18 (20.7)	2 (8.7)	16 (25)	
*4: Disagree*	18 (20.7)	6 (26.1)	12 (18.8)	
*5: Strongly disagree*	18 (20.7)	7 (30.4)	11 (17.2)	
Day care on site available, *n* (%)	71 (81.6)	19 (82.6)	52 (81.3)	0.885
Evaluation of available day care, *n* (%)				0.045
*Satisfactory*	12 (16.9)	1 (5.3)	11 (21.2)	
*Mostly satisfactory*	19 (26.8)	3 (15.8)	16 (30.8)	
*Mostly unsatisfactory*	19 (26.8)	5 (26.3)	14 (26.9)	
*Unsatisfactory*	21 (29.6)	10 (52.6)	11 (21.2)	
Participation in support programme, *n* (%)				0.121
*Yes*	5 (5.7)	3 (13.0)	2 (3.1)	
*None offered*	67 (77)	18 (78.3)	49 (76.6)	
*Not interested*	15 (17.2)	2 (8.7)	13 (20.3)	

### Impact of pregnancy on career

**Figure 5:**
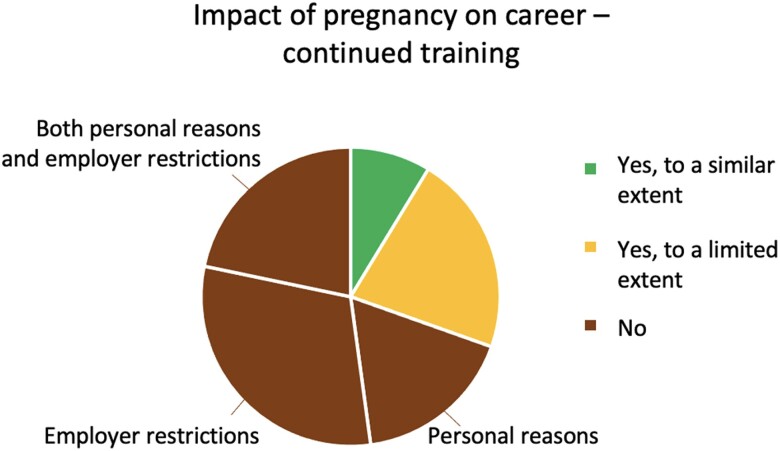
Impact of pregnancy on career. Was continued training and pursuit of career goals possible during pregnancy?.

During pregnancy, 21.7% of women felt limited and 69.6% unable to continue to pursue their career goals and training. The most common reason reported was restrictions by employers (75.0%), personal limitations were reported by 56.3% (*Figure 5*). 68.2% of women were not allowed to continue with invasive procedures, although 90.9% stated they had wanted to. 73.9% of women were not allowed to work with radiation, although 78.3% stated they had wanted to. Only 21.7% of women reported there was a dedicated policy or programme for pregnant interventionalists at their institution. Aspects assessed regarding pregnancy are shown in detail in the Supplementary material.

## Discussion

Along with the growing demand on health care services and research in many fields of medicine, including cardiac EP, there is an increasing acknowledgement of the role of female electrophysiologists.^4,20–22^ At the same time, the awareness of gender inequities, especially in leading positions and scientific workforces, is also increasing.^1–3^ In an attempt to understand and overcome gender inequities, larger surveys have investigated on general topics of concern to female physicians in cardiology training.^8,11,12,15,18^ Our study is the first to specifically analyse factors hampering women with a dedicated career goal of invasive EP to pursue their careers further and successfully compete with men. We succeeded to assemble the voices of female and male physicians in Germany who had been at similar starting points of their careers before parenthood, and with similar career goals.

The main findings of the study are the following. The majority of both women and men with a career goal of invasive EP before parenthood achieved this goal (80.0% and 96.4%, respectively). However, women changed their career paths to a higher extent compared with men after parenthood. They achieved fewer qualifications, academic degrees and leading positions, independent of age, while they were in charge for most of the childcare at home. Most women and men felt supported by their families, but most women did not feel supported by their employers in terms of work–family conflicts. Satisfactory day care for children and support programmes offered by employers were scarce. The majority of women suffered from working restrictions during pregnancy hampering their careers.

Our results suggest that existing role models and the distribution of childcare at home are a major factor influencing the career paths of female physicians in invasive EP. However, lack of flexibility at work was perceived at least equally important compared to lack of flexibility at home, by most women. The majority of women did not feel supported by their employer. This presents an important point, as support of employers and supervisors is known to play a crucial role in the motivation of women in terms of career.^23–26^

Two more specific points raised in this study were a lack of satisfactory day care offered by employers and a lack of specific support programmes that might allow female physicians to work more effectively on their careers while reducing overall working hours for childcare at the same time. The potential of onsite childcare offered by the employer has been propagated since decades, but increasingly since the pandemic, and does provide a promising tool in terms of work–family compatibility for medical workers in general.^27–32^ Organizational interventions for advancing women in leadership have likewise been propagated and were shown to motivate women to pursue their careers more ambitiously. These may include mentorship or networking programmes, different kinds of fundings, educational, and research support.^23,24,33–40^ Our study adds to this body of evidence, as our study population consisted of physicians who had participated in dedicated educational EP programmes. As an overall positive result, the majority of women in this study population had remained active in invasive EP despite parenthood, and the percentage of female consultants is relatively large compared to the general EP community in Germany.^3^ However, a minority of study participants benefitted from additional family or gender-specific support by their employers—a factor that should be addressed, together with the deficient day care. Specific career promoting efforts may serve to compensate for some of the gender associated disadvantages and family associated disadvantages such as working restrictions during pregnancy or reduced working hours during parenthood, which may also apply for men, supporting shared caregiving duties.

Working restrictions during pregnancy were faced by most female study participants. This point is especially relevant considering the generally long training time in invasive EP.^21,37,41^ 73.9% of women were not allowed any radiation exposure during pregnancy, which is an even larger number compared with the one reported in a recent European survey (48.1%).^15^ While working restrictions may apply for several appropriate reasons including infectious risk and physical impairment, neither the German, nor most other country’s national maternity protection legacies, nor a current consensus document of the European cardiac societies, justify general bans from radiation exposure for pregnant workers.^42^ Moreover, occupational radiation exposure in invasive EP can be minimized up to zero-fluoroscopy using modern 3D mapping systems.^43–46^ While many female physicians still seem to be concerned about radiation exposure and are not aware of the existing EHRA consensus document,^15^ there is an increasing awareness of the safety of low radiation exposure under protective measures.^47^ In fact, following this trend in awareness, the majority of women within our study population were willing to be exposed to radiation under protective measures during pregnancy, and changes in career goals were hardly attributed to radiation concerns. However, education of employers seems to lack behind and needs to be addressed.

We believe the results of our study primarily comfort and motivate women with a career goal in invasive EP, as most women were actually able to follow their goals despite parenthood and were overall satisfied with their work. Invasive EP, in contrast to other branches of invasive cardiology, hardly requires night or weekend shifts and therefore has the potential to be more compatible with family responsibilities. Moreover, occupational radiation exposure is much lower compared with other branches of interventional cardiology.^43,48^ However, there is still much room for improvement, as women in invasive EP were still less successful, had fewer children and were less satisfied with their achievements compared with men.

While gender stereotypes are shifting very gradually,^2,49–51^ our survey, together with other studies, confirms there is a remaining unequal distribution of childcare among academic males and females, which seems a major factor for gender inequities, including those in invasive EP.^12,52^ Regarding this, increasing awareness continues to be crucial in the further pursuit of more gender equity. Moreover, we suggest addressing more specific measures on the employers’ side that are currently deficient, including the revision of regulations for pregnant workers and the increased implementation of supportive programmes and children day care, to support women in EP.

### Limitations

This study has the limitation of being a national study from Germany, and not all results may apply to other regions. However, as previous studies have shown, the overall issues are similar across different countries. Another limitation is a selection bias due to the very carefully selected study population, derived from participants of exclusive fellowship programmes for aspiring young doctors at the beginning of their EP careers. Our results may not apply for a larger majority of female physicians or cardiology fellows. However, it was the explicit goal of the current study to specifically address aspiring women that already have chosen invasive EP as their career goal in order to identify more specific problems in this population and evaluate possible effective measures that may bring about change for women in EP. We believe many aspects of our results similarly apply to other fields of cardiology.

## Conclusions

Women had to change their career paths more often than men after parenthood but the majority was still able to continue with an invasive EP career. Despite a similar starting point of both genders before parenthood, women were able to accomplish fewer professional achievements and were less satisfied with their work. A fundamental reason seems to be the distribution of childcare at home, but also a lack of flexibility and support by employers. Specifically, attention should be drawn to deficiencies in day care offerings, lack of specific support programmes as well as often unnecessary working restrictions during pregnancy to further support women in EP.

## Lead author biography


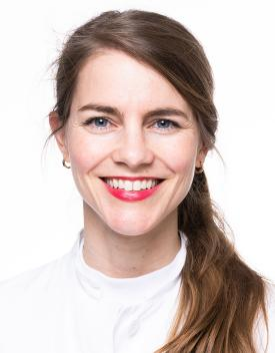


Dr Johanna Mueller-Leisse (MD, MSc) is a consultant cardiologist specialized in electrophysiology and devices at Hannover Heart Rhythm Center, Hannover Medical School, Germany. She has two children and has benefitted from daycare onsite, educational and research support.

## Supplementary Material

oeae070_Supplementary_Data

## Data Availability

All data underlying the article are available at Hannover Medical School and will be shared on reasonable request to the corresponding author.
