# Supplementary material for: Reasons for gender inequities in invasive electrophysiology: a survey on family issues and career paths of female and male electrophysiology fellows in Germany
Source: Eur Heart J Open. 2024 Aug 20;4(5):oeae070. doi: 10.1093/ehjopen/oeae070 (PMC11382543; doi:10.1093/ehjopen/oeae070)
Supplement: oeae070_Supplementary_Data [file oeae070_supplementary_data.docx]

**Appendix**

Questions

1. Are we allowed to use your answers for analysis and publication?
2. How old are you?
3. What is your gender?
   1. Male
   2. Female
   3. Diverse
4. Please state all your current qualifications:
   1. Cardiology specialist
   2. Internal Medicine specialist
   3. Other specialist
   4. Consultant
   5. Head of department
   6. Special Rhythmology of DGK – A (invasive EP)
   7. Special Rhythmology of DGK – B (devices)
   8. Other certifications of DGK
   9. Medical doctor (“Dr. med.”) academic title
   10. “Privatdozent” (PD) academic title
   11. Professor
   12. Head of research group
5. What kind of institution are you working in?
   1. University hospital
   2. Non-university hospital
   3. Private practice
6. How many hours do you work (in percent of full-time)?
   1. 100%
   2. 75-99%
   3. 50-74%
   4. <50%
   5. 0
7. Are you working interventionally (multiple answers)?
   1. Yes, in interventional electrophysiology
   2. Yes, in device surgery
   3. Yes, in interventional cardiology
   4. No
8. How many interventions have you performed on your own?
   1. 0
   2. 1-9
   3. 10-49
   4. 50-99
   5. >100
   6. ≥500
9. How many pubmed-listed first or last authorships do you have?
   1. 0
   2. 1-4
   3. 5-9
   4. 10-14
   5. ≥15
10. How satisfied are you with your work (Likert scale)?
    1. Very satisfied
    2. Satisfied
    3. Undecided
    4. Unsatisfied
    5. Very unsatisfied
11. How many children do you have?
12. Have you already continued to work since parental leave?
13. How old were you when you had your first child?
14. What kind of institution were you working in at the time?
    1. University hospital
    2. Non-university hospital
    3. Private practice
15. How many hours (in percentage of full-time) were you working before parenthood?
16. Please state all qualifications that applied before parenthood
    1. Cardiology fellow
    2. Cardiology specialist
    3. Internal Medicine specialist
    4. Other specialist
    5. Consultant
    6. Head of department
    7. Special Rhythmology of DGK – A (invasive EP)
    8. Special Rhythmology of DGK – B (devices)
    9. Other certifications of DGK
    10. Medical doctor (“Dr. med.”) academic title
    11. “Privatdozent” (PD) academic title
    12. Professor
    13. Head of research group
17. How many interventions had you performed on your own before parenthood?
    1. 0
    2. 1-9
    3. 10-49
    4. 50-99
    5. >100
    6. ≥500
18. How many pubmed-listed first or last authorships did you have before parenthood?
    1. 0
    2. 1-4
    3. 5-9
    4. 10-14
    5. ≥15
19. Please state all career goals that you had before parenthood
    1. Interventional cardiology
    2. Interventional electrophysiology
    3. Research career
    4. Other:
20. To what extent did you change your career goals after parenthood (Likert scale)?
    1. No changes
    2. Little changes
    3. Intermediate changes
    4. Strong changes
    5. Very strong changes
21. What were relevant reaons for changes in your career goals after parenthood?
    1. Changes in personal priorities (Likert scale)
       1. Strongly agree
       2. Agree
       3. Undecided
       4. Disagree
       5. Strongly disagree
    2. Changes in personal interests/preferences (Likert scale i-v)
    3. Lack of support by employer / flexibility at work (Likert scale i-v)
    4. Lack of support by family / flexibility at home (Likert scale i-v)
    5. Radiation exposure conderns (Likert scale i-v)
    6. Financial aspects (Likert scale i-v)
22. How many months of parental leave did you take for your first child?
23. How many hours (in percent of full-time) have you been working, in average, since parenthood?
24. How did your interventional activity change after parenthood?
    1. No (more) interventional activity
    2. Less interventional activity
    3. Similar interventional activity
    4. More interventional activity
25. How did your research activity change after parenthood?
    1. No (more) research activity
    2. Less research activity
    3. Similar research activity
    4. More research activity
26. Do you agree or disagree to the following: “I felt supported by my employer / supervisors In terms of work-familie issues”.
    1. Strongly agree
    2. Agree
    3. Undecided
    4. Disagree
    5. Strongly disagree
27. Was the most relevant person for this question male or female?
    1. Male
    2. Female
    3. Diverse
28. What applies to day care support by your employer (e.g. on site day care)?
    1. Satisfactory
    2. Mostly satisfactory
    3. Mostly unsatisfactory
    4. Unsatisfactory
    5. None available
29. What applies to dedicated support programs / fundings / scholarships for women or parents?
    1. I participated in such a program and it was helpful.
    2. I participated in such a program but it was not helpful.
    3. I did not have the opportunity to participate in such a program.
    4. I did not participate for other reasons (e.g. not interested)
30. Do you agree or disagree to the following: “I felt supported by my family In terms of work-familie issues”.
    1. Strongly agree
    2. Agree
    3. Undecided
    4. Disagree
    5. Strongly disagree
31. Please indicate in % the distribution of childcare at home
    1. Myself:
    2. My partner:
    3. Family/friends
    4. Babysitter/au pair/other
32. In the year(s) following parental leave, how many days were you absent from work on average (per year) for childcare (e.g. sick child, day care closings)?
33. The following 7 questions apply to pregnancy. Please continue if you have had a pregnancy, and end the survey otherwise.
    1. Continue
    2. End of survey
34. During your (first) pregnancy, how many days were you absent from work apart from maternity leave / protection (e.g. sick, occupational ban)?
35. What applies to interventional activity during your pregnancy (multiple answers)?
    1. I was not allowed to continue with interventional work.
    2. I did not want to continue with interventional work.
    3. Not applicable (e.g. no interventional work before pregnancy)
    4. I reduced interventional activity.
    5. I continued to a similar extent with interventional activity.
    6. I worked mostly with “zero fluoroscopy”
36. What applies to device surgery during your pregnancy (multiple answers)?
    1. I was not allowed to continue doing device implantations.
    2. I did not want to continue doing device implantations.
    3. Not applicable (e.g. no device implantations before pregnancy)
    4. I reduced device implantations.
    5. I continued to a similar extent with device implantations.
37. What applies to research activity during your pregnancy (multiple answers)?
    1. I did not do research before or during pregnancy.
    2. I reduced research during pregnancy.
    3. I did research to a similar extent during pregnancy.
    4. I increased research activity during pregnancy.
38. What applies to radiation exposure during your pregnancy (multiple answers)?
    1. I was not allowed radiation exposure.
    2. I did not want to be exposed to radiation.
    3. Not applicable (e.g. no exposure before pregnancy)
    4. I reduced radiation exposure.
    5. I was exposed to a similar extent.
39. Was there any dedicated policy / program / guideline for pregnant interventionalists at your institution?
    1. Yes
    2. No
    3. I don’t know
40. During your pregnancy, were you able to further pursue your training and career goals?
    1. Yes, to a similar extent.
    2. Yes, but to a limited extent.
    3. No, mainly for personal / health reasons.
    4. No, mainly because of restrictions by my employer / supervisor.
    5. No, both reasons apply.

Supplementary table 1: Reasons for changes of career goals after parenthood.

|  | All | Women | Men | p-value |
| --- | --- | --- | --- | --- |
| Changes in personal prirorities, median (range) | 2 (1-4) | 1 (1-4) | 2 (1.4) | 0.725 |
| - 1: Strongly agree | 33 (47.1) | 11 (52.4) | 22 (44.9) |  |
| - 2: Agree | 28 (40.0) | 7 (33.3) | 21 (42.9) |  |
| - 3: Undecided | 5 (7.1) | 1 (4.3) | 4 (8.2) |  |
| - 4: Disagree | 4 (5.7) | 2 (9.5) | 2 (4.1) |  |
| - 5. Strongly disagree | 0 | 0 | 0 |  |
| Changes in personal interests, median (range) | 3 (1-5) | 3 (1-5) | 3 (1-5) | 0.725 |
| - 1: Strongly agree | 9 (12.9) | 2 (9.5) | 7 (14.3) |  |
| - 2: Agree | 18 (25.7) | 6 (28.6) | 12 (24.5) |  |
| - 3: Undecided | 15 (21.4) | 3 (14.3) | 12 (24.5) |  |
| - 4: Disagree | 16 (22.9) | 3 (14.3) | 13 (26.5) |  |
| - 5. Strongly disagree | 12 (17.1) | 7 (33.3) | 5 (10.2) |  |
| Lack of flexibility at work, median (range) | 3 (1-5) | 2 (1-5) | 3 (1-5) | 0.056 |
| - 1: Strongly agree | 17 (24.3) | 7 (33.3) | 10 (20.4) |  |
| - 2: Agree | 14 (20.0) | 6 (28.6) | 8 (16.3) |  |
| - 3: Undecided | 14 (20.0) | 4 (19) | 10 (20.4) |  |
| - 4: Disagree | 21 (30.0) | 3 (14.3) | 18 (36.7) |  |
| - 5. Strongly disagree | 4 (5.7) | 1 (4.8) | 3 (6.1) |  |
| Lack of flexibility at home, median (range) | 3 (1-5) | 2 (1-5) | 3 (1-5) | 0.136 |
| - 1: Strongly agree | 10 (14.3) | 5 (23.8) | 5 (10.2) |  |
| - 2: Agree | 20 (28.6) | 8 (38.1) | 12 (24.5) |  |
| - 3: Undecided | 11 (15.7) | 1 (4.8) | 10 (20.4) |  |
| - 4: Disagree | 16 (22.9) | 3 (14.3) | 13 (26.5) |  |
| - 5. Strongly disagree | 13 (18.69 | 4 (19) | 9 (18.4) |  |
| Radiation exposure concerns, median (range) | 5 (1-5) | 4 (1-5) | 5 (1-5) | 0.100 |
| - 1: Strongly agree | 4 (5.7) | 1 (4.8) | 3 (6.1) |  |
| - 2: Agree | 6 (8.6) | 4 (19) | 2 (4.1) |  |
| - 3: Undecided | 5 (7.1) | 3 (14.3) | 2 (4.1) |  |
| - 4: Disagree | 13 (18.6) | 3 (14.3) | 10 (20.4) |  |
| - 5. Strongly disagree | 42 (60.0) | 10 (47.6) | 32 (65.3) |  |
| Financial reasons, median (range) | 4 (1-5) | 4 (1-5) | 3 (1-5) | 0.206 |
| - 1: Strongly agree | 9 (12.9) | 2 (9.5) | 7 (14.3) |  |
| - 2: Agree | 17 (24.3) | 4 (19) | 13 (26.5) |  |
| - 3: Undecided | 6 (8.6) | 1 (4.8) | 5 (10.2) |  |
| - 4: Disagree | 12 (17.1) | 4 (19) | 8 (16.3) |  |
| - 5. Strongly disagree | 26 (37.1) | 10 (47.6) | 16 (32.7) |  |

Supplementary table 2: Impact of pregnancy on career.

|  |  |
| --- | --- |
| Continuing to follow career goals during pregnancy, n (%) |  |
| - Yes, to a similar extent | 2 (8.7) |
| - Yes, to a limited extent | 5 (21.7) |
| - No | 16 (69.6) |
| Reasons for limitations, n (%) |  |
| - Mainly personal | 4 (25.0) |
| - Mainly restrictions by employer | 7 (43.8) |
| - Both apply | 5 (31.2) |
| Interventios during pregnancy, n (%) |  |
| - Not allowed | 15 (68.2) |
| - Not willing to | 2 (9,1) |
| - Yes, to a smaller extent | 4 (18.2) |
| - Yes, to a similar extent | 3 (13.6) |
| Surgery during pregnancy, n (%) |  |
| - Not allowed | 11 (55.0) |
| - Not willing to | 3 (15.0) |
| - Yes, to a smaller extent | 4 (20.0) |
| - Yes, to a similar extent | 2 (10.0) |
| Radiation exposure during pregnancy, n (%) |  |
| - Not allowed | 17 (73.9) |
| - Not willing to | 5 (21.7) |
| - Yes, to a smaller extent | 2 (8.7) |
| - Yes, to a similar extent | 2 (8.7) |
| Available policy or program for pregnant workers |  |
| - Yes | 5 (21.7) |
| - No | 15 (65.2) |
| - Not known | 3 (13.1) |
| Research acitivty during pregnancy |  |
| - Not applicable | 6 (26.1) |
| - Yes, to a smaller extent | 5 (21.7) |
| - Yes, to a similar extent | 12 (52.2) |
| Days off work during pregnancy for health reasons (aside from maternity leave) |  |
| - Missing during entire pregnancy, n (%) | 2 (12.5) |
| - Days missing, median (range) | 2 (0-126) |
